# Up-Regulating ERIC by CRISPR-dCas9-VPR Inhibits Cell Proliferation and Invasion and Promotes Apoptosis in Human Bladder Cancer

**DOI:** 10.3389/fmolb.2021.654718

**Published:** 2021-03-29

**Authors:** Jiangeng Yang, An Xia, Huajie Zhang, Qi Liu, Hongke You, Daoyuan Ding, Yonghua Yin, Bo Wen

**Affiliations:** Department of Urology, Shenzhen Hospital of Integrated Traditional Chinese and Western Medicine, Shenzhen, China

**Keywords:** CRISPR-dCas9-VPR, long non coding RNA, ERIC, cancer, bladder

## Abstract

LncRNAs are defined as non-coding RNAs that are longer than 200 nucleotides in length. The previous studys has shown that lncRNAs played important roles in the regulation of gene expression and were essential in mammalian development and disease processes. Inspired by the observation that lncRNAs are aberrantly expressed in tumors, we extracted RNA from Bladder urothelial carcinoma and matched histologically normal urothelium from each patient and bladder carcinoma cell lines. Then, we reversed transcribed them into cDNA.Last, we investigated the expression patterns of ERIC by the fluorescence quantitative PCR in bladder cancer tissues and cell lines. CRISPR-dCas9-VPR targeting ERIC plasmid was transfected into T24 and 5637 cells, and cells were classified into two groups: negative control (NC) and ERIC overexpression group. MTT assay, transwell assay, and flow cytometry were performed to examine changes in cell proliferation, invasiveness, and apoptosis. We found that the expression of ERIC was down-regulated in bladder urothelial carcinoma compared to matched histologically normal urotheliam. The differences of the expression of this gene were large in the bladder cancer lines. Compared with the negative control group, the ERIC overexpression group showed significantly decreased cell proliferation rate (*t* = 7.583, *p* = 0.002; *t* = 3.283, *p =* 0.03) and invasiveness (*t* = 11.538, *p* < 0.001; *t* = 8.205, *p =* 0.01); and increased apoptotic rate (*t* = −34.083, *p* < 0.001; *t* = −14.316, *p* < 0.001). Our study lays a foundation for further study of its pathogenic mechanism in bladder cancer.

## Introduction

Long non-coding RNA (lncRNAs) is an RNA molecule with a length greater than 200bp. It has mrna-like structure and polyA tail and promoter structure after splicing. During differentiation, it has dynamic expression and different splicing modes. LncRNAs are mainly transcribed by RNA polymerase II (RNA PII), polyadenylation and splicing, and most of them are located in the nucleus (Zhang et al., 2013). LncRNAs do not encode proteins, but regulate gene expression in the form of RNA at the epigenetic, transcriptional and post-transcriptional levels (Mattick, 2005).

At first, researchers found that they do not encode proteins, so they were treated as the "noise" of genome transcription, with no biological function (Gershon, 2005). With the deepening of studies on lncRNAs, the regulatory mechanism of lncRNAs in cells has gradually attracted extensive attention. At present, the functions of most of the lncRNAs have not been clearly studied, but most scholars believe that the study of lncRNAs will help to understand the complex gene regulatory network of living organisms, and is expected to provide new molecular basis for the prediction, diagnosis and treatment of complex diseases (Zhang et al., 2013).

Numerous studies have shown that the expression level of certain lncRNAs can be significantly changed in tumor tissues and cells. For example, pcat-1 and PCGEM1 are significantly down-regulated in prostate cancer (Gibb et al., 2011; Prensner et al., 2011). While in breast cancer, GAS5 is overexpressed (Mourtada-Maarabouni et al., 2009). This change may be closely related to many biological processes, so lncRNAs can provide a basis and target for the molecular diagnosis and treatment of malignant tumors. With the rapid development of high-throughput screening sequencing technology and bioinformatics, more lncrnas with important functions in tumors will be discovered, which will also provide new theories and basis for the diagnosis and treatment of these major diseases.

ERIC, e2f1-regulated Inhibitor of Cell Death, is a long non-coding RNA–XLOC 006942. The gene is regulated by E2Fs transcription and inhibits apoptosis induced by E2F1 and DNA damage. On the chromosome diagram, ERIC (also known as TCONS_00014875) is located on chromosome 8 (CHR 8:141646242–141648531), corresponding to a position on band 8q24.3, and transcribed from the positive chain. ERIC is composed of two exons and a transcription sequence with a size of 1745 bp (Feldstein et al., 2013).

Bladder cancer is the most common malignant tumor in the genitourinary system, which is mainly divided into three subtypes: transitional cell carcinoma (TCC), squamous cell carcinoma (SCC) and adenocarcinoma. Its main pathological type is TCC, accounting for more than 90% of cases of bladder cancer (Rahmani et al., 2013). Among them, 70–80% of patients were diagnosed as non-muscular invasive bladder cancer (formerly known as superficial bladder cancer), and the other 20–30% were diagnosed as muscular invasive bladder cancer.

The incidence of bladder cancer ranks 9th among all malignant tumors and 6th among male tumors. Globally, there are 350,000 new cases a year (Griffiths, 2013). In the United States, there are 72,570 new cases of bladder cancer and 15,210 new deaths each year (Siegel et al., 2013). It costs $40 billion a year in the United States alone to treat bladder cancer, which requires repeated examinations and follow-up treatment after surgery. Thus, bladder cancer is the most expensive malignancy in the United States on average per patient. In China, the incidence and mortality of bladder cancer are the highest in urinary system tumors. In the past decade, the incidence of the disease has been increasing year by year and getting younger (Li et al., 2017). The most common treatment for bladder cancer is surgery, chemotherapy, immunotherapy, and radiation (Liu et al., 2012). Many patients were already in the middle and late stage when they went to the doctor. Bladder cancer, like other cancers, has become a major public health problem threatening human health.

Currently, the pathogenesis of bladder cancer is still not clear. However, with the development of molecular biology and genetic technology in recent years, more and more scholars believe that the pathogenesis of bladder cancer is a complex process involving multiple factors, multiple genes and multiple steps. Accumulation of abnormal mutant genotypes and the role of external oncogenic environment eventually led to the emergence of malignant phenotype of bladder cancer. It is an urgent task to explore the pathogenesis of bladder cancer and find specific targets for bladder cancer, so as to achieve early diagnosis, effective treatment and prevent its recurrence.

Currently, some international scholars have proposed the hypothesis of abnormal chromatin remodeling in tumors, including bladder cancer (Michaeleen, 2011). Most studies of long non-coding RNAs have recognized that they regulate the expression of many genes mainly through large region chromatin remodeling (Costa, 2008). So LncRNAs are important regulators of the interaction between gene expression and the important pathways of cell growth, proliferation, differentiation and survival. Changes in the function of lncRNAs promote tumor formation and development, as well as metastasis of prostate cancer, bladder cancer and renal cell carcinoma. LncRNAs can be used as non-invasive tumor markers in malignant tumors of the urinary system. The increasing study on the molecular mechanism of LncRNAs in normal and malignant cells will contribute to a better understanding of tumor biology and may be a new therapeutic target for the treatment of cancer of the urinary system (Chung et al., 2011; Luo et al., 2013; Yang et al., 2013; Martens-Uzunova et al., 2014). Therefore, lncRNAs with significant expression differences in bladder cancer were screened out to further explore their biological functions, which will bring new opportunities for the diagnosis, treatment and postoperative detection of this disease.

## Methods

### Sample Collection

A total of 5 bladder cancer samples were obtained. The 4 normal samples were defined as bladder tissues located 2.0 cm outside of visible cancer lesions. All resection samples were confirmed to be bladder cancer by clinical pathology. The collection and use of the patient samples were reviewed and approved by the Institutional Ethics Committee of Shenzhen Shajing Affiliated Hospital of Guangzhou Medical University, and written informed consent from all patients was appropriately obtained.

### Cell Culture

The bladder cancer cell lines T24 and 5,637 were purchased from the Institute of Cell Research, Chinese Academy of Sciences, Shanghai, China. These cells were cultured in DMEM medium which had added 10% fetal bovine serum, and were grown in the 37°C atmosphere which contains 5% CO_2_.

### Total RNA Preparation and Reverse Transcription

Total RNA was extracted from tissue samples, and cell lines using TRIZOL (Invitrogen, United States) according to the manufacture’s protocol and evaluated using Agilent 2,100 Bioanalyzer (Agilent Technologies, United States). RT was carried out using Omniscript Reverse Transcriptase kit (Qiagen, Hilden, Germany). The total reaction volume was 20 μl including 1 μg RNA. The reaction mixture was incubated at 42°C for 60 min, heated at 95°C for 10 min and then cooled on ice. The reaction was diluted 1:1 with water and aliquoted for further analysis.

### qRT-PCR Assay

The TRIzol reagent (Invitrogen, Carlsbad, CA, United States) was used to extract the total RNAs from the cancer tissues and bladder cancer cell lines. The cDNAs were synthesized from the total extracted RNAs with the RevertAid™ First Strand cDNA Synthesis Kit (Fermentas, Hanover, MD, United States). The All-in-One™ qPCR Mix (GeneCopoiea Inc., Rockville, MD, United States) was used to carry out the qRT-PCR assay in our study. ERIC forward primer:5′- AGC​CTG​TGG​CTA​CCT​CCT​TT-3′; reverse primer:5′- CTT​GCA​CCC​ATA​TGC​AGA​CA-3′; GAPDH forward primer:5′-CGCTCTCTGCTCCTCCTGTTC-3′; reverse primer:5′-ATCCGTTGACTCCGACCTTCAC-3′.

### Plasmid Transfection

The CRISPR-dCas9-VPR targeting ERIC plasmid used in this experiment was purchased from Gima Company. After transforming into competent *Escherichia coli* DH5alpha, the plasmids were extracted by monoclone and sequenced to verify the cloned plasmids. At the logarithmic growth stage, cells were transfected into 24-well plates to adjust the cell density to 4 × 10^5^/well; the fusion degree of cells was 70 –80%. In accordance with the Lipofectamine 2,000 transfection instructions, the plasmids were transfected into T24 and 5,637. The cells were cultured in a constant-temperature incubator with 5% CO_2_ at 37°C. After 48 h, the culture plates were taken out and the cells were collected for subsequent experiments.

### MTT Assay

Cells in logarithmic growth phase were cultured overnight in an incubator in 5% CO_2_ at 37°C for 4 h after adjusting the cell density to 5 × 10^4^ cells/mL. The cells were seeded in a 96-well plate in 100 μL of cell suspension and then cultured in a constant-temperature incubator with 5% CO_2_ at 37°C for 4 h. After incubation, 0.15 mL DMSO was added and the suspension was shaken for 10 min. Optical density at 568 nm (OD_568_) was measured using a microplate reader.

### Transwell Assay

After 48 h of successful transfection, the cells of each group were digested, collected with 0.25% trypsin, and centrifuged. The cells were then washed twice with pre-cooled phosphate-buffered saline (PBS). Cells were suspended in a serum-free medium and counted by the plate count method. Next, 0.8 mL medium with 10% FBS was transferred into a 24-well plate, which was then placed in a transwell chamber. Then 1 mg/mL matrigel (100 µL) was added vertically to the bottom of the upper transwell chamber. After the matrigel solidified, 200 mL cell suspension was added to the upper transwell chamber and cultured in 5% CO_2_ at 37°C for 24 h. The transwell was then removed; the chamber was washed with PBS; and the cells were fixed in 10% MeOH. After 30 min, the membrane was removed; the cells were subjected to crystal violet (0.5%) staining at room temperature for 20 min and finally washed with PBS. Images were acquired and cell numbers calculated under a microscope.

### Flow Cytometry

After 2 days of culture, cells were subjected to trypsin digestion (0.25%) and then collected in a special flow tube. Approximately 10^5^ suspended cells were centrifuged. Detection was conducted following the instructions of the Annexin V-APC/7-AAD detection kit. Binding buffer (0.05 mL, 5 × 10^5^/mL) was added to cells, and cells were resuspended. The 7-AAD solution (5 μL) was added, and cells were incubated for 15 min at room temperature. Finally, 0.45 mL binding buffer and 1 µL Annexin V-APC were added for reaction at room temperature in the dark for 15 min. The samples were investigated through flow cytometry.

### Statistical Analysis

Statistical analyses were performed using SPSS (v. 22.0). Data were presented as mean ± SD, and independent t-tests were used to investigate intergroup distinctions. A *p* < 0.05 was considered significant.

## Results

### PCR Results of Bladder Cancer and Paracancer Tissues

**FIGURE 1:**
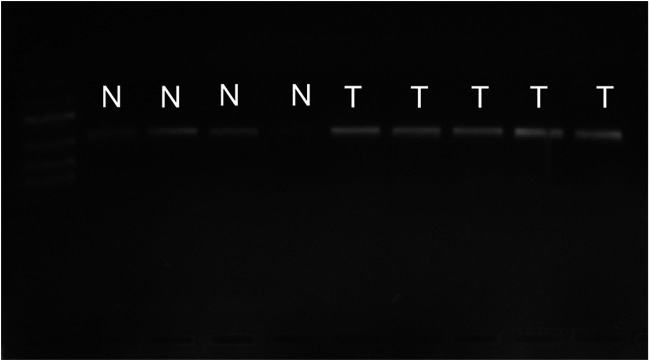
PCR results of cancer tissues and adjacent tissues. Bands of PCR in both the cancerous tissue and the paracancer tissueN, normal bladder tissurs. T, bladder tumor tissues.

As shown in Figure 1, there were bands of PCR in both the cancerous tissue and the paracancer tissue, indicating the success of reverse transcription. However, compared with bands of bladder cancer, the normal bands were lighter in brightness, indicating that the amount of cDNA in reverse transcription was less. Fluorescence quantitative PCR was performed.

### General PCR Results of Bladder Cancer Cell Lines T24 and 5,637

**FIGURE 2:**
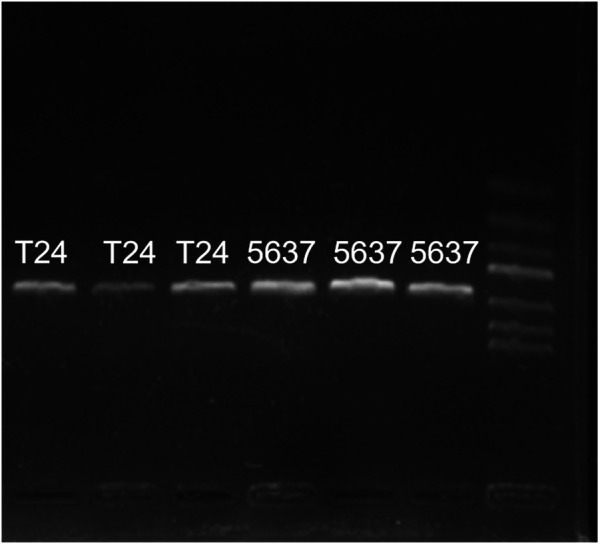
General PCR results of cell lines T24 and 5,637 cDNA. PCR bands were observed in both cell lines T24 and 5,637, while the band brightness of the three groups of 5,637 was lighter than that of T24.

Common PCR results of cell lines T24 and 5,637 were shown in Figure 2: PCR bands were observed in both cell lines T24 and 5,637, indicating successful reverse transcription. However, the band brightness of the three groups of 5,637 cell lines was lighter than that of T24. Fluorescence quantitative PCR was performed.

### Expression of ERIC Gene in Bladder Cancer

**FIGURE 3:**
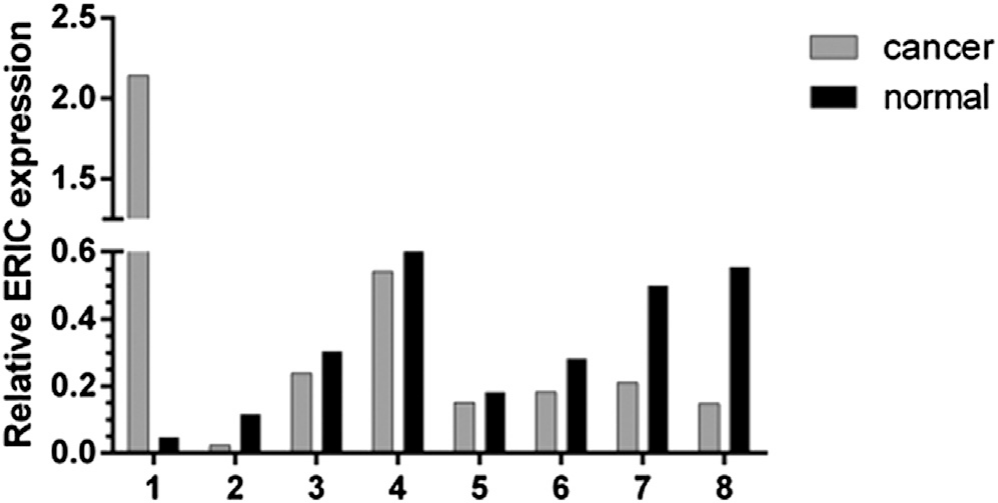
Expression of ERIC gene in bladder cancer tissues and paracancer tissues of 8 patients. The relative expression level of ERIC was determined by qRT-PCR assay. The mean value was shown.

Real-time fluorescence quantitative PCR was used to detect the expression level of ERIC gene in 36 bladder cancer tissues and para-cancer normal tissues. As shown in Figure 3, the expression level of ERIC in bladder cancer tissue was higher in seven cases than that in the adjacent group (about 2.48 times on average), and only one case was lower than that in the adjacent group.

### Expression of ERIC Gene in Cell Lines T24 and 5,637

**FIGURE 4:**
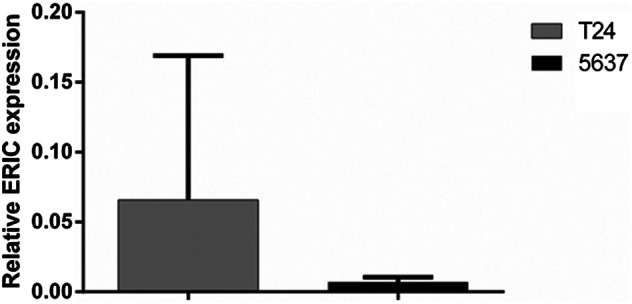
Expression of ERIC gene in bladder cancer cell lines T24 and 5,637. The relative expression level of ERIC was determined by qRT-PCR assay. The mean value ± SD was shown.

The fluorescence quantitative PCR results of cell lines T24 and 5,637 were shown in Figure 4: the expression level of T24 was significantly higher than that of 5,637.

### Effect of ERIC on T24 and 5,637 Cell Proliferation

**FIGURE 5:**
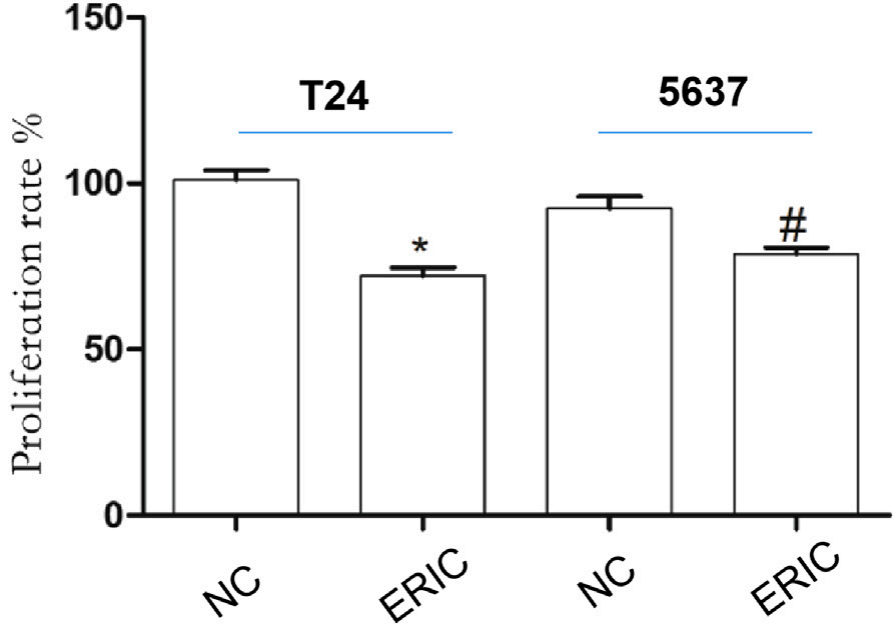
Changes in cell proliferation rate in each group. The proliferation rate was determined by MTT assay. The mean value ± SD was shown. ^*^ *p* < 0.05. ^#^ *p* < 0.05.

Compared with that in the T24 NC group, cell proliferation rate in the T24 ERIC group was significantly decreased (*t* = 7.583, *p* < 0.05). Similarly, compared with that in the 5637 NC group, the cell proliferation rate in the 5637 ERIC group was significantly decreased (*t* = 3.283, *p* < 0.05) (Figure 5).

### Effect of ERIC on T24 and 5,637 Cell Invasion

**FIGURE 6:**
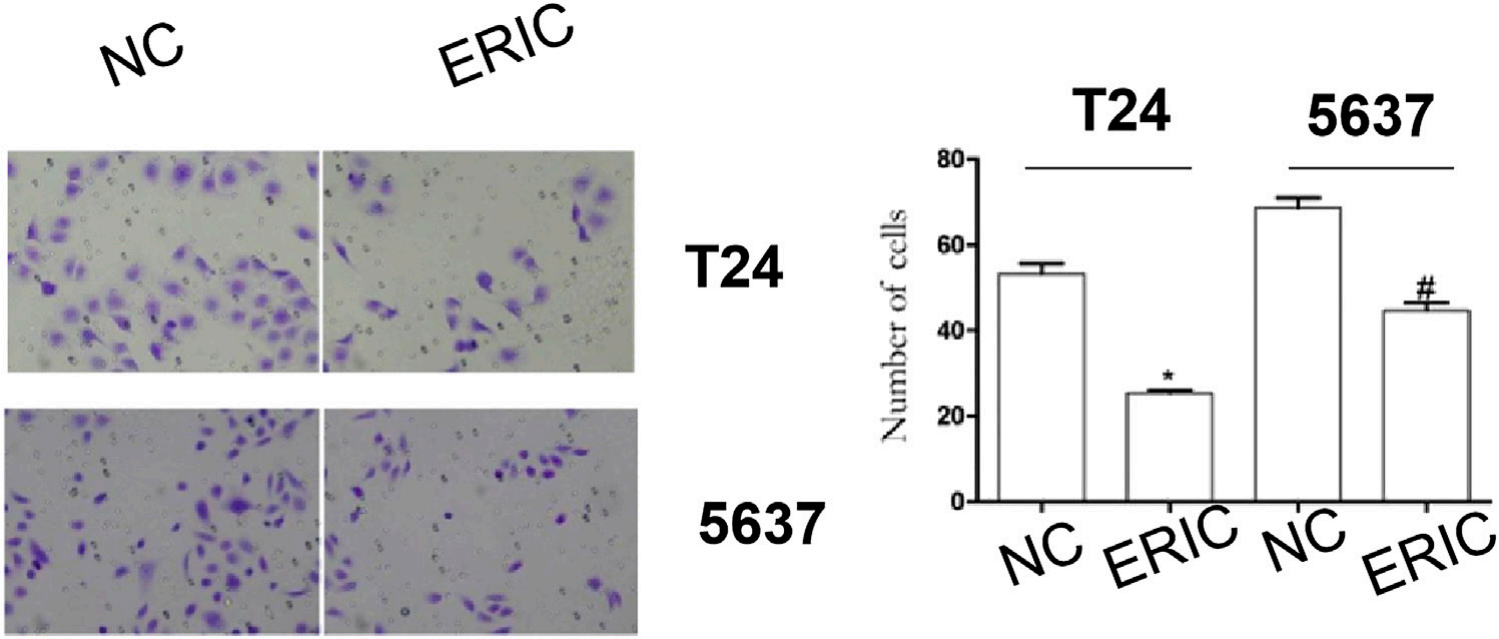
Changes in cell invasion levels in each group (200 times). Cell invasion level was determined by transwell assay. The mean value ± SD was shown. ^*^ *p* < 0.05. ^#^ *p* < 0.05.

Compared with that in the ERIC groups (T24 and 5,637 + ERIC), the number of cells in the negative control groups (T24 and 5637 NC) was significantly increased (*t* = 11.538, *p* < 0.001; *t* = 8.205, *p* < 0.05, respectively) (Figure 6).

### Effects of ERIC Inhibition on T24 and 5,637 Cell Apoptosis

**FIGURE 7:**
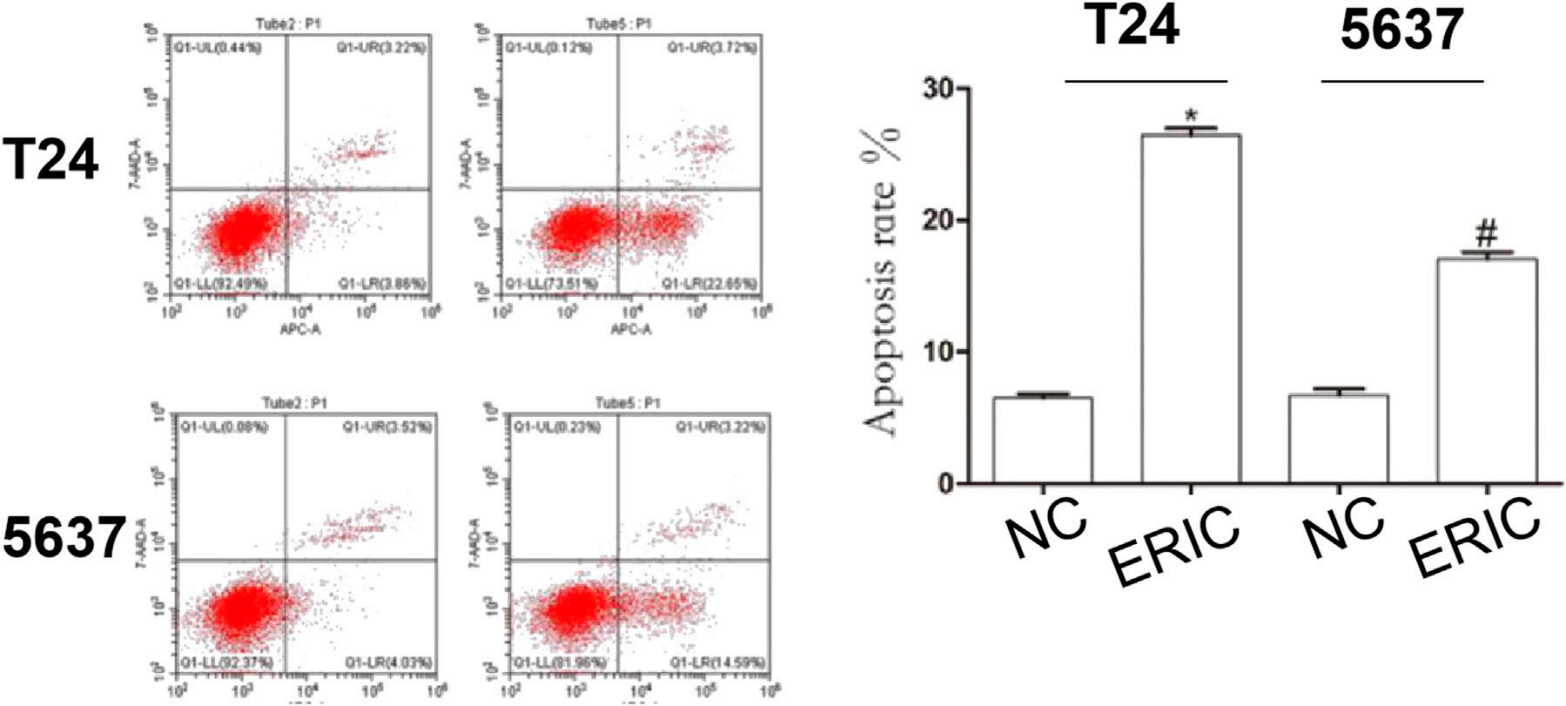
Changes in apoptotic rate in each group. Cell apoptosis level was determined by flow cytometry. The mean value ± SD was shown. ^*^ *p* < 0.05. ^#^ *p* < 0.05.

To study the influence of ERIC on BCC apoptosis, flow cytometry was used to detect T24 and 5,637 cell apoptosis. Compared with that in the T24 NC group, the apoptosis rate in the T24 ERIC group was dramatically increased (*t* = −34.083, *p* < 0.001). Compared with that in the 5637 NC group, the apoptosis rate in the 5637 ERIC group was also increased (*t* = −14.316, *p* < 0.001) (Figure 7).

## Discussion

In China, bladder cancer is the first malignant tumor of the urinary system (Li et al., 2017). Currently, smoking and occupational exposure to aromatic amine are the main risk factors for bladder cancer (Bosetti et al., 2011). The occurrence and development of bladder cancer is a multi-stage and multi-step evolutionary process, and the long-term accumulation of abnormal genotypes leads to the emergence of malignant phenotypes. The purpose of determining the expression status of certain genes in bladder cancer is to determine whether this gene is related to bladder cancer, so as to lay a foundation for the follow-up study on its function and molecular mechanism. Finally, we hope to find specific targets for bladder cancer and provide a new molecular basis for our clinical diagnosis, treatment and prognosis.

Long non-coding RNA (lncRNA) is a class of RNA molecules with a transcript length of more than 200 nt, which does not itself encode proteins or has little function of encoding proteins, and is generally transcribed in mammalian genomes. It was originally thought to be a by-product of the transcription process, with no biological function. With the deepening of research, new lncrnas have been continuously discovered, and more and more evidence shows that lncrnas have complex biological functions and are closely related to human diseases, especially the occurrence of tumors (Gupta et al., 2010).

The long non-coding RNA ERIC transcript is 1745bp in size and is directly regulated by E2F in a p53 independent manner (Feldstein et al., 2013). The expression of ERIC gene is regulated by cell cycle and reaches its peak in G1 phase. Studies have shown that ERIC gene and transcription factor E2F1 constitute a negative feedback pathway, which regulates the activity of E2F1, and ERIC inhibits the cell apoptosis induced by E2F1. With the increase of E2F1 activity, the expression of ERIC gene also increased.

In this study, this gene was down-regulated in bladder cancer tissues compared with para-cancer tissues, suggesting that this gene may play a role of “tumor suppressor gene”. As they are regulated by transcription factors E2F1 and E2F3, these two genes are also down-regulated in bladder cancer. It laid a solid foundation for further research on the function of this gene.

Currently, it has become one of the difficult problems in urology surgery to find specific targets for bladder cancer, explore new treatment methods and overcome the shortcomings of traditional treatment methods such as chemotherapy and surgery. Revealing the expression and function of lncRNAs in bladder cancer is an effective way to find specific targets. Xue M et al. found that the up-regulated expression of lncrna-uca1 can increase the proliferation, migration and invasion ability of cells and inhibit apoptosis under the hypoxia condition (Xue et al., 2014); Fan Y et al. demonstrated a positive correlation between the expression of lncrna-uca1 and Wnt6 *in vivo*, and ultimately demonstrated that UCA1 increased cisplatin resistance in bladder cancer cells by enhancing the expression of Wnt6 (a member of the Wnt6 pterless MMTV integration site family 6), thus identifying potential targets for overcoming chemotherapy resistance (Fan et al., 2014a); Fan Y TGF–such as beta MALAT1 induced bladder cancer cells and epithelial mesenchymal (EMT), MALAT1 excessive expression was significantly associated with bladder cancer patients with low survival rate, the expression of targeted inhibit MALAT1 and suz12 can inhibit the TGF - beta induced cell migration, and invasion characteristics, and think the MALAT1 inhibition may be used to inhibit the development of bladder cancer is a promising treatment (Fan et al., 2014b). These studies will provide theoretical basis for the research and development of new ways and new drugs to treat bladder cancer.

At present, there are few studies on ERIC gene, and its function in tumor has not been reported. Although it was found to be down-regulated in bladder cancer in this study, it was not statistically significant due to the small number of study samples. Thererfore we further study the function of this gene to reveal its role in bladder cancer. CRISPR-dCas9-VPR technology was a new approach for upregulating celluar gene expression without affecting cell viabillity. By binding to the promoter region of targeted gene, it could activate lncRNA transcription (Kardooni et al., 2018; Zhang et al., 2018; Blanas et al., 2019). A CRISPR-dCas9-VPR targeting ERIC plasmid was constructed and transfected into bladder cancer cell lines T24 and 5,637 to detect the effect on proliferation, invasion, apoptosis and other aspects of bladder cancer cell lines and reveal its function. Multi-level research will be helpful to find specific targets for bladder cancer, and provide new scientific basis for clinical targeted treatment of this disease and the research and development of new drugs. In our study, the proliferation and migration of ERIC positive cells were significantly reduced, and cell apoptosis was increased.

In conclusion, downregulated ERIC can inhibit the invasion of human bladder cancer, and promote their apoptosis.

## Data Availability

The original contributions presented in the study are included in the article/Supplementary Material, further inquiries can be directed to the corresponding authors.
